# The Clinical Practice and Best Aerosol Delivery Location in Intubated and Mechanically Ventilated Patients: A Randomized Clinical Trial

**DOI:** 10.1155/2021/6671671

**Published:** 2021-04-03

**Authors:** Chuanlin Zhang, Jie Mi, Zeju Zhang, Xueqin Wang, Yunxiao Zhu, Xinyi Luo, Ruiying Gan, Xiaoya Chen, Yujun Zou

**Affiliations:** ^1^Department of Critical Care Medicine, The First Affiliated Hospital of Chongqing Medical University, Chongqing, China; ^2^School of Nursing, Chongqing Medical and Pharmaceutical College, Chongqing, China; ^3^The Second Affiliated Hospital of Army Medical University, Chongqing, China

## Abstract

This randomized clinical trial (RCT) is aimed at exploring the best nebulizer position for aerosol delivery within the mechanical ventilation (MV) circuitry. This study enrolled 75 intubated and MV patients with respiratory failure and randomly divided them into three groups. The nebulizer position of patients in group A was between the tracheal tube and Y-piece. For group B, the nebulizer was placed at the inspiratory limb near the ventilator water cup (80 cm away from the Y-piece). For group C, the nebulizer was placed between the ventilator inlet and the heated humidifier. An indirect competitive enzyme-linked immunosorbent assay (ELISA) was used to measure salbutamol drug concentrations in serum and urine. The serum and urine salbutamol concentrations of the three groups were the highest in group B, followed by group C, and the lowest in group A. Serum and urine salbutamol concentrations significantly differed among the three groups (*P* < 0.05). It was found that the drug was statistically significant between group differences for groups B and A (*P* = 0.001; *P* = 0.002, respectively) for both serum and urine salbutamol concentrations. There were no significant differences observed among the other groups. It was found that the drug concentrations were the highest when the nebulizer was placed 80 cm away from the Y-piece, while the location between the tracheal tube and the Y-piece with the higher frequency of nebulizer placement was the location with the lowest drug concentration.

## 1. Introduction

Invasive mechanically assisted ventilation is a common treatment for intensive care unit (ICU) patients [1]. About 3 million patients in the United States of America (USA) received tracheal intubation at the emergency or ICU departments. Each year, one-third of patients require mechanical ventilation (MV) for more than 48 h, and many patients require aerosol therapy during the MV [2]. Aerosol therapy is a safe and convenient method of treatment and commonly used in patients with invasive MV in the ICU, especially for patients with asthma and chronic obstructive pulmonary disease [3, 4]. The three most commonly used aerosolizing drugs are bronchodilators, corticosteroids, and antibiotics [5, 6]. However, the effect of aerosolized inhalation is reduced due to the establishment of an artificial airway in a tracheal intubated patient [7, 8]. Macintyre and colleagues first reported that, in patients with artificial airways, aerosol transmission was only one-sixth of what it was in patients without artificial airways [9]. Over the past 25 years, with the development of aerosol equipment and operation technology, the aerosol delivery to invasive MV patients has almost been matched and even exceeded that reported in patients with nonartificial airways [10–12].

Many factors may affect the efficacious delivery of aerosols to the lungs. These factors are associated with patients, drugs, devices, artificial airways, ventilator settings, and ventilator circuits [13–18]. The position of nebulizers placed by ICU nurses is another important factor. In clinical practice, the most common nebulizer position was between the tracheal tube and the Y-piece (41~46%) or after Y-piece (39~41%), respectively [5, 19]. Many in vitro tests showed that, when the nebulizer was placed after Y-piece or between the ventilator inlet and heated humidifier, drug delivery to the lungs was the largest [20–23]. However, there have been fewer in vivo experiments about how different nebulizer positions affect aerosol delivery. Morarine found no difference in urine drug concentrations in urine between aerosolized drugs delivered via Y-piece and the inspiratory limb closed to heated humidifier [24].

The authors knew that in clinical practice, the common nebulizer position was between the tracheal tube and the Y-piece [5, 19]. However, it is not known whether the higher frequency of atomizer placement is the location with the highest drug concentration. Therefore, upon review of clinical practice and in vitro literature, three locations were selected (between the tracheal tube and the Y-piece, at the inspiratory limb near the ventilator water cup (80 cm away from Y-piece) and between the ventilator inlet along with a heated humidifier). Serum and urine drug concentrations were used as outcomes. It was hypothesized that placing the nebulizer in position between the tracheal tube and the Y-piece would result in higher urinary and drug salbutamol concentrations.

## 2. Methods

### 2.1. Study Design

This study design was an RCT investigating the effects of different nebulizer positions on drug concentrations. Informed written consent was obtained from all participants or their responsible family member. Ethical approval was obtained from the Ethics Committee of the First Affiliated Hospital of Chongqing Medical University (Document No. 2019-311).

### 2.2. Patients in the Study

This RCT was performed between June 2019 and January 2021. Eligible patients were aged ≥18 years with a previous diagnosis of respiratory failure with invasive MV (intubation) admitted to the ICU at the First Affiliated Hospital of Chongqing Medical University and were prescribed salbutamol. Exclusion criteria were patients with (1) no indwelling catheter; (2) do not agree to participate in this study; (3) systolic blood pressure < 100 mmHg or receiving adrenaline booster drugs; (4) renal dysfunction (i.e., serum creatinine concentration > 2 mg/dL or urine output < 50 ml/h); (5) myocardial infarction, unstable angina or arrhythmia, and refractory hypoxemia; and (6) those allergic to salbutamol drugs, or patients had used salbutamol drugs 7 days prior to inclusion. Seventy-five random numbers were chosen through the random number table method, and each number was divided by 3, and if the remainder was 0, it was assigned to group A; if the remainder was 1, it was assigned to group B; if the remainder was 2, it was assigned to group C. When the patient who met the inclusion criteria, a random number was randomly selected from an envelope and was assigned to group X by the study designer. Research designer was not involved in data collection and analysis.

### 2.3. Study Procedure

**Figure 1:**
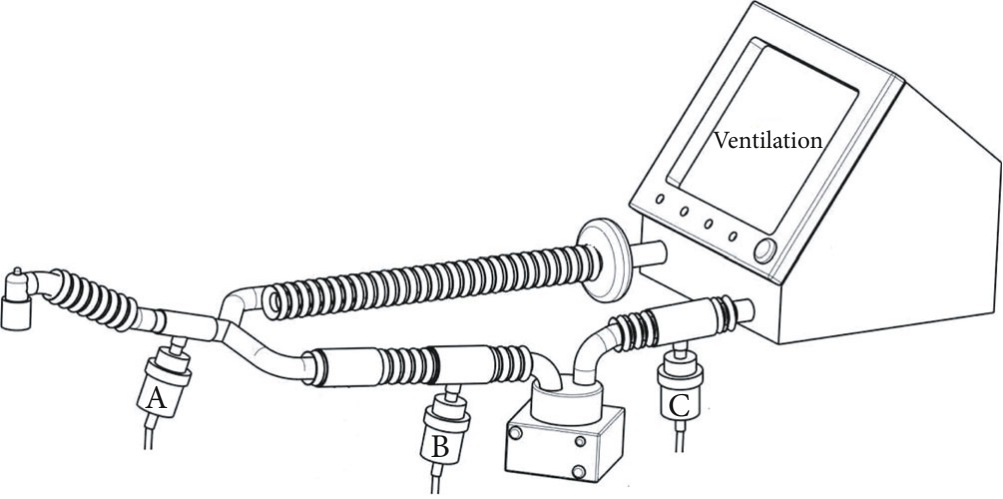
The position of the nebulizer in the three groups are after Y-piece (position A), inspiratory limbs near the ventilator water cup (80 cm away from Y-piece) (position B), and between the ventilator inlet and the heated humidifier (position C).

Seventy-five (75) patients who met the inclusion criteria were selected, and the power of this study was 86.2%. These individuals were randomly divided into three groups. The nebulizer position of patients in group A was between the tracheal tube and the Y-piece. For group B, the nebulizer was positioned at the inspiratory limb near the ventilator water cup (80 cm away from Y-piece). For group C, the nebulizer was between the ventilator inlet along with a heated humidifier (Figure 1).

There were many indicators for evaluating the efficacy of nebulization inhalation, such as chest tightness, wheezing, airway resistance, lung compliance, and drug concentrations. Drug concentrations in blood and urine samples from patients were chosen because these measures were both objective and easy to measure. Preparation before the study: (1) the patients were positioned in a semirecumbent position with the bed head raised by 30 degrees, (2) fully suctioned of endotracheal and airway secretions, (3) the humidifier was turned off, (4) the atomizer was tapped regularly until the atomization was completed, and (5) the nebulizer was washed with sterile water.

The study was completed by five trained researchers. At 9 : 30 every morning, 3 ml of normal saline and 2.5 ml (5 mg) of salbutamol were added to the Jet atomizer (Emedical). The nebulizer's mass median aerodynamic diameter (MMAD) was 3.6 *μ*m, and geometric standard deviation (GSD) was 2.2 *μ*m. All patients received “Dräger” ventilator- (a ventilator with fog function-) assisted ventilation. Before nebulization, the researchers ensured that the patient had not been treated with salbutamol within 7 days. The doctor uniformly adjusted the ventilator parameters to assist-control ventilation (A-CV) mode, with a tidal volume of 500 ml, PEEP of 5 cmH_2_O, breathing frequency of 16 times/minute, and inspiratory/exhalation = 1/2. Ten minutes after the inhalation of salbutamol, 5 ml of venous blood was taken (using a heparin-free anticoagulant tube), and 10 ml of urine was retained after 30 minutes and stored in a -80°C refrigerator by researchers [21, 25, 26].

### 2.4. Data Collection

An indirect competitive ELISA was used to measure salbutamol drug concentrations in serum and urine. The specific steps were as follows: ① Centrifuge 0.5 ml of the urine or serum sample at 4,000 x g for 5 minutes and use 50 ul of the supernatant per well for the assay. ② Add 50 *μ*l of each salbutamol standards in duplicate into different wells. 50 *μ*l was added of each sample in duplicate into different sample wells. Then, 100 *μ*l of antibody #1 was added and the solution mixed well by gently rocking the plate manually for 1 minute. ③ The plates were incubated for 30 minutes at room temperature (22.5 ± 2.5) °C and then washed three times with 250 *μ*l of 1x wash solution. After the last wash, the plate was inverted, and the plate gently tapped dry on paper towels. ④ 150 *μ*l of 1× HRP-conjugated antibody #2 was added, and the plate was incubated for 30 minutes at room temperature (22.5 ± 2.5) °C. After this, the plate was washed three times with 250 *μ*l of 1× wash solution. After the last wash, the plate was inverted and gently tapped dry onto paper towels. ⑤ Then, 100 *μ*l of TMB substrate was added, after which the reaction was immediately timed. The solution was mixed by gently rocking the plate manually for 1 minute while incubating. After incubating for 15 minutes at room temperature (22.5 ± 2.5) °C, 100 *μ*l of stop buffer was added to stop the enzyme reaction. ⑥ The plate was read as soon as possible following the addition of the stop buffer using a plate reader with a 450 nm wavelength.

The patient's airway symptoms were assessed using the patient airway symptom scoring tool. Patients were evaluated according to sputum aspiration, sputum volume, sputum color, sputum consistency, cough irritation, and lung rhonchus, with scores of 0 ~ 3 for each indicator. The total scores were 0 ~ 18 points [27].

### 2.5. Study Outcomes

The primary outcomes included salbutamol drug concentrations in serum and urine associated with nebulizer placement at each of the three locations. Demographic characteristics, APACHE-II, tracheal tube size, ICU length of stay, MV time, airway average pressure, airway peak pressure, and airway clinical symptom score were also collected.

### 2.6. Quality Control of the Study

Prior to the study, five researchers were uniformly trained. Patients were strictly screened according to the inclusion and exclusion criteria. The specimens were accurately retained and stored by researchers. This meant that 10 minutes after the inhalation of salbutamol, 5 ml of venous blood was taken (using a heparin-free anticoagulant tube), 10 ml of urine was retained after 30 minutes, and the specimen was stored in a -80°C refrigerator. The researchers who conducted the clinical trial only participated in the data collection, and the data were analyzed by others. Salbutamol drug concentrations in serum and urine were tested by the same researcher.

### 2.7. Statistical Analysis

All data were analyzed using SPSS (version 21; IBM Corp, Armonk, New York, USA). General data were expressed as mean ± standard deviation, percentage, or median (quartile). General data comparisons among the three groups were performed using a chi-square analysis, analysis of variance (ANOVA), or nonparametric tests, as appropriate. The blood and urine concentrations of salbutamol in each of the three nebulizer position groups were compared using ANOVA, and the pairwise comparisons were performed using the LSD method. Statistical significance was set at *P* < 0.05.

## 3. Results

### 3.1. General Information about Eligible Patients

**Figure 2:**
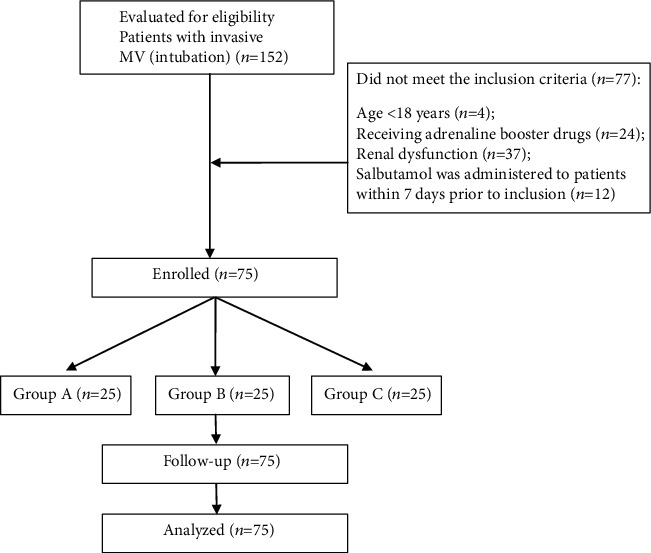
Consort flow diagram of this study procedure.

A total of 152 patients were screened, of which 75 met the inclusion criteria and were randomly assigned to 3 groups of 25 patients each. Finally, the data of 75 patients was analyzed (Figure 2). The enrolled 75 patients (43 males and 32 females) aged 22 to 89 years (57.3 ± 17.1 years). The mean body mass index was 21.7 ± 2.9, APACHE II was 18.9 ± 7.0, and the mean airway clinical symptom score was 10.4 ± 3.0. The median and interquartile range for the length of ICU stay was 5 (3–9) days and 56 (28–102) h for the duration of MV. There was no statistical difference in the baseline data of the three groups (Table 1).

### 3.2. Salbutamol Levels in Serum and Urine

The salbutamol serum and urine concentrations of the three groups were the highest in group B, followed by group C, and were the lowest in group A. There was a significant difference in the 3 groups about the serum and urine salbutamol concentration (*P* < 0.05). Further analysis using the LSD test found that there were statistically significant difference between group B and group A (*P* = 0.001; *P* = 0.002, respectively) both in serum and urine salbutamol concentrations, while there were no significant differences among other groups (Table 2).

## 4. Discussion

This RCT indicated that when the nebulizer was placed 80 cm away from the Y-piece, the salbutamol concentrations were the highest in both serum and urine, whilst the lowest drug concentration was found when the nebulizer was located between the tracheal tube and Y-piece. This is completely contrary to the author's hypothesis and means that the common nebulizer location is not the optimal position when ICU nurses implement aerosol therapy for intubated and MV patients.

Many factors may affect aerosol delivery for patients with invasive MV, and the nebulizer position is one such crucial factor [28]. In the clinical practice, ICU nurses often place the nebulizer between the tracheal tube and the Y-piece or after Y-piece. Ehrmann et al. [5, 19] found that 41~46% of physicians would place the nebulizer between the tracheal tube or after the Y-piece in 39~41% of patients. However, placements in other positions only accounted for 10~20%. A previous study also showed that the nebulizer was usually placed at between the tracheal tube and Y-piece and after the Y-piece [29]. However, in this study, the lowest drug concentration was found between the tracheal tube and Y-piece, a frequently placed location.

In vitro experiments showed that, when the nebulizer was placed in different locations, aerosol delivery changed in both adult and pediatric lung models [20, 22, 23, 30–33]. Many studies indicated that, when the nebulizer was placed at either the ventilator or humidifier, aerosol delivery was the largest. The less efficient was received when nebulizer was placed at Y-piece or between Y-piece and the tracheal tube [20, 22, 23, 30, 33]. As reported by Air et al. [30], the jet nebulizer was the most efficient in position 15 cm from the ventilator under both nonhumidified and heated humidified conditions, while when the heating humidifier is turned off, the position between Y-piece and the tracheal tube was the lowest. This RCT differed in part from these studies. Although there was no statistically significant difference between positions B and C, drug concentrations were slightly higher in position B than that in position C. In this study, the position B where the nebulizer was placed 80 cm away from the Y-piece was the optimal position. This might be related to the fact that the nebulizer was connected to the inspiratory limb away from the artificial airway. The pipeline could therefore store mist and increase aerosol delivery [34]. Similar to the above findings, the aerosol delivery was the lowest at the position between Y-piece and the tracheal tube. However, the limitation of these studies was that these were conducted in vitro. In vivo studies were needed to draw definitive conclusions [35].

To the authors' knowledge, no in vivo experiments have attempted to detect salbutamol concentrations relative to nebulizer positioning after the Y-piece, at the inspiratory limb near the ventilator water cup, and between the ventilator inlet and the heated humidifier at the same time. Moraine et al. [24] divided 38 patients with invasive MV into 2 groups: the nebulizer positioned before the heated humidification system or the inspiratory limb before the Y-piece. The authors found that the urine concentrations of ipratropium bromide did not differ between the 2 groups. This is different from this study, which may be due to the fact that the nebulizer is too close to the Y-piece to store mist, and that part of the aerosol is easy to be lost from the expiratory limb. In this study, the nebulizer was placed 80 cm away from the Y-piece. Indeed, other study reported when the nebulizer was placed at 30 cm from the Y-piece, the aerosol transport efficiency was higher than that at Y-piece [22]. Another reason may be that different drugs are studied, which can affect the results [36].

### 4.1. Clinical Implications

Most patients with invasive MV will receive aerosol therapy every day, and ICU nurses play a crucial role during the implementation of this therapy. However, this present study showed marked discrepancies in the nebulizer operation between trial and clinical practice paradigms. This was particularly true when it came to the optimal nebulizer position. Thus, targeted atomization education or training is necessary for all ICU nurses. Increasing the awareness of ICU nurses to different nebulizer positions will likely affect aerosol delivery and help determine which position is best for patients. Moreover, standards or guidelines for aerosol therapy should focus on standardizing the atomization operation and developing measures to deal with potential hazards [37]. Furthermore, targeted atomization educational programs should be implemented through departments or hospital education and academic conferences. Finally, future atomization studies should attempt to mirror clinical practice settings and be easy to operate. Even though the study data shows that changing the ventilator parameters during the atomization operation may be more effective, this may be very difficult to implement in clinical practice [5].

### 4.2. Limitations

There are several limitations to this study. First, the drug concentrations were not measured in the three different positions on one patient, but instead randomly placed the nebulizer on different patients. Some patients receive invasive MV for fewer than three days. If the time interval is too short, some salbutamol may remain in the patient. However, when comparing the baseline data of the three groups, it was found that the groups' baseline data were similar and likely did not affect the study results. Second, clinical measures were not measured; thus, whether receiving a bit more of salbutamol was better for the patients was unknown. Finally, only three nebulizer locations were selected because these three positions were the ones that were most often reported in the literature and were ones commonly placed by ICU nurses.

## 5. Conclusion

In conclusion, serum and urine drug concentrations were used as outcomes to determine which placement was the best among three locations (between the tracheal tube and the Y-piece, at the inspiratory limb near the ventilator water cup (80 cm away from Y-piece), and between the ventilator inlet along with a heated humidifier). It was found that the drug concentrations were the highest when the nebulizer was placed 80 cm away from the Y-piece while the location between the tracheal tube and the Y-piece with the higher frequency of nebulizer placement was the location with the lowest drug concentration.

## Figures and Tables

**Table 1:** General information pertaining to patients in the study.

Characteristics	Group A^∗^ (*n* = 25)	Group B^∗^ (*n* = 25)	Group C^∗^ (*n* = 25)	*t* / *F* / *x*^2^	*P*
Sex					
Male	17 (39.5%)	11 (25.6%)	15 (34.9%)	3.052	0.217
Female	8 (25.0%)	14 (43.8%)	10 (31.3%)		
Age, y	55.5 ± 16.3	59.5 ± 18.3	56.8 ± 17.1	0.342	0.711
BMI	21.7 ± 3.2	21.6 ± 3.3	21.8 ± 2.3	0.037	0.964
APACHE II	16.8 ± 6.7	20.5 ± 7.0	19.6 ± 7.2	1.941	0.151
Tracheal tube size	7.4 ± 0.3	7.4 ± 0.2	7.3 ± 0.4	0.308	0.736
ICU length of stay, d	5 (1.8, 21.7)	5 (3.4, 14.0)	5 (4.3, 26.6)	0.264	0.876
Mechanical ventilation, h	62 (45, 107)	66 (54,168)	50 (46, 192)	0.418	0.811
Airway average pressure, cmH_2_0	10.1 ± 1.8	10.7 ± 2.6	10.7 ± 2.9	0.409	0.666
Airway peak pressure, cmH_2_0	19.4 ± 3.9	21.0 ± 5.5	19.0 ± 4.9	1.226	0.299
Airway clinical symptom score [27]	10.2 ± 2.6	10.4 ± 3.1	10.7 ± 3.5	0.188	0.829

^∗^Group A, the nebulizer was between the tracheal tube and the Y-piece. Group B, the nebulizer was positioned at the inspiratory limb near the ventilator water cup (80 cm away from Y-piece). And group C, the nebulizer was between the ventilator inlet along with a heated humidifier.

**Table 2:** Salbutamol excretion in blood and urine at different nebulization locations.

Characteristics	A (*n* = 25)	B (*n* = 25)	C (*n* = 25)	*F*	*P*
Salbutamol concentrations in serum	1.41 ± 0.45^∗^	1.94 ± 0.57^∗^	1.64 ± 0.63	5.887	0.004
Salbutamol concentration in urine	0.47 ± 0.23^∗^	0.70 ± 0.31^∗^	0.59 ± 0.24	5.085	0.009

^∗^The LSD method showed that the excretion of salbutamol in blood and urine was statistically different in group A and group B (*P* = 0.001; *P* = 0.002, respectively).

## Data Availability

The data used to support the findings of this study are available from the corresponding author upon request.

## References

[1] Tobin M., Manthous C. (2017). Mechanical ventilation. *American Journal of Respiratory and Critical Care Medicine*.

[2] Lovett P. B., Flaxman A., Stürmann K. M., Bijur P. (2006). The insecure airway: a comparison of knots and commercial devices for securing endotracheal tubes. *BMC Emergency Medicine*.

[3] Luyt C. E., Hékimian G., Bréchot N., Chastre J. (2018). Aerosol therapy for pneumonia in the intensive care unit. *Clinics in Chest Medicine*.

[4] Pleasants R. A., Hess D. R. (2018). Aerosol delivery devices for obstructive lung diseases. *Respiratory Care*.

[5] Ehrmann S., Roche-Campo F., Sferrazza Papa G. F., Isabey D., Brochard L., Apiou-Sbirlea G. (2013). Aerosol therapy during mechanical ventilation: an international survey. *Intensive Care Medicine*.

[6] Dugernier J., Ehrmann S., Sottiaux T. (2017). Aerosol delivery during invasive mechanical ventilation: a systematic review. *Critical Care*.

[7] Dhand R., Guntur V. P. (2008). How best to deliver aerosol medications to mechanically ventilated patients. *Clinics in Chest Medicine*.

[8] Dhand R. (2017). How should aerosols be delivered during invasive mechanical ventilation?. *Respiratory Care*.

[9] Macintyre N. R., Silver R. M., Miller C. W., Schuler F., Coleman R. E. (1985). Aerosol delivery in intubated, mechanically ventilated patients. *Critical Care Medicine*.

[10] Dhand R., Tobin M. J. (1997). Inhaled bronchodilator therapy in mechanically ventilated patients. *American Journal of Respiratory and Critical Care Medicine*.

[11] Dhand R. (2005). Inhalation therapy with metered-dose inhalers and dry powder inhalers in mechanically ventilated patients. *Respiratory Care*.

[12] Dhand R., Sohal H. (2008). Pulmonary drug delivery system for inhalation therapy in mechanically ventilated patients. *Expert Review of Medical Devices*.

[13] Dhand R. (2008). Aerosol delivery during mechanical ventilation: from basic techniques to new devices. *Journal of Aerosol Medicine and Pulmonary Drug Delivery*.

[14] Wang L., Guan C., Qin X., Qu Y. (2018). Effects of aerosol inhalation on respiratory mechanical parameters under different ventilation patterns and ventilator parameters. *Chinese Journal of Critical Care Medicine*.

[15] Miller D. D., Amin M. M., Palmer L. B., Shah A. R., Smaldone G. C. (2003). Aerosol delivery and modern mechanical ventilation: in vitro/in vivo evaluation. *American Journal of Respiratory and Critical Care Medicine*.

[16] Vecellio L., Guérin C., Grimbert D., de Monte M., Diot P. (2005). In vitro study and semiempirical model for aerosol delivery control during mechanical ventilation. *Intensive Care Medicine*.

[17] Ari A., Fink J. B., Dhand R. (2012). Inhalation therapy in patients receiving mechanical ventilation: an update. *Journal of Aerosol Medicine and Pulmonary Drug Delivery*.

[18] Guerin C., Fassier T., Bayle F., Lemasson S., Richard J. C. (2008). Inhaled bronchodilator administration during mechanical ventilation: how to optimize it, and for which clinical benefit?. *Journal of Aerosol Medicine and Pulmonary Drug Delivery*.

[19] Ehrmann S., Roche-Campo F., Bodet-Contentin L. (2016). Aerosol therapy in intensive and intermediate care units: prospective observation of 2808 critically ill patients. *Intensive Care Medicine*.

[20] Ari A., Areabi H., Fink J. B. (2010). Evaluation of aerosol generator devices at 3 locations in humidified and non-humidified circuits during adult mechanical ventilation. *Respiratory Care*.

[21] ElHansy M. H. E., Boules M. E., el Essawy A. F. M. (2017). Inhaled salbutamol dose delivered by jet nebulizer, vibrating mesh nebulizer and metered dose inhaler with spacer during invasive mechanical ventilation. *Pulmonary Pharmacology & Therapeutics*.

[22] Berlinski A., Willis J. R. (2013). Albuterol delivery by 4 different nebulizers placed in 4 different positions in a pediatric ventilator in vitro model. *Respiratory Care*.

[23] Dugernier J., Wittebole X., Roeseler J. (2015). Influence of inspiratory flow pattern and nebulizer position on aerosol delivery with a vibrating-mesh nebulizer during invasive mechanical ventilation: an in vitro analysis. *Journal of Aerosol Medicine and Pulmonary Drug Delivery*.

[24] Moraine J. J., Truflandier K., Vandenbergen N., Berré J., Mélot C., Vincent J. L. (2009). Placement of the nebulizer before the humidifier during mechanical ventilation: Effect on aerosol delivery. *Heart & Lung*.

[25] Duarte A. G., Dhand R., Reid R. (1996). Serum albuterol levels in mechanically ventilated patients and healthy subjects after metered-dose inhaler administration. *American Journal of Respiratory and Critical Care Medicine*.

[26] Marik P., Hogan J., Krikorian J. (1999). A comparison of bronchodilator therapy delivered by nebulization and metered- dose inhaler in mechanically ventilated patients. *Chest*.

[27] Wen M., Zheng F. M., Li G. X., Xu J. Q. (2011). Observation on the airway effect of nebulizer at different positions for patients undergoing mechanical ventilation. *Chinese General Nursing*.

[28] Fink J., Ari A. (2013). Aerosol delivery to intubated patients. *Expert Opinion on Drug Delivery*.

[29] Zhang C. L., Mi J., Wang X. Q. (2021). Knowledge and current practices of ICU nurses regarding aerosol therapy for patients treated with invasive mechanical ventilation: a nationwide cross-sectional study. *Journal of Clinical Nursing*.

[30] Ari A., Atalay O. T., Harwood R., Sheard M. M., Aljamhan E. A., Fink J. B. (2010). Influence of nebulizer type, position, and bias flow on aerosol drug delivery in simulated pediatric and adult lung models during mechanical ventilation. *Respiratory Care*.

[31] Lyu S., Li J., Yang L. (2020). The utilization of aerosol therapy in mechanical ventilation patients: a prospective multicenter observational cohort study and a review of the current evidence. *Annals of Translational Medicine*.

[32] Thomas S. H., O'Doherty M. J., Page C. J., Treacher D. F., Nunan T. O. (1993). Delivery of ultrasonic nebulized aerosols to a lung model during mechanical ventilation. *The American Review of Respiratory Disease*.

[33] Anderson A. C., Dubosky M. N., Fiorino K. A., Quintana V., Kaplan C. A., Vines D. L. (2017). The effect of nebulizer position on aerosolized epoprostenol delivery in an adult lung model. *Respiratory Care*.

[34] Respiratory therapy group (2014). Expert consensus on atomization inhalation during mechanical ventilation (draft). *Chinese Journal of Tuberculosis and Respiratory Diseases*.

[35] Dhanani J., Fraser J. F., Chan H. K., Rello J., Cohen J., Roberts J. A. (2016). Fundamentals of aerosol therapy in critical care. *Critical Care*.

[36] Berlinski A. (2020). Innovation in aerosol drug delivery during adult mechanical ventilation. *Respiratory Care*.

[37] Laube B. L., Janssens H. M., de Jongh F. H. (2011). What the pulmonary specialist should know about the new inhalation therapies. *The European Respiratory Journal*.

